# Annual Registry for Hepatitis C and Hepatitis B is Needed for Predicting the Burden of Hepatocellolar Carcinoma in Iran

**DOI:** 10.5812/hepatmon.8089

**Published:** 2013-02-24

**Authors:** Zeinab Fazeli, Mohsen Vahedi, Sara Ashtari, Mohamad Amin Pourhoseingholi

**Affiliations:** 1 Proteomics Research Center, Students' Research Committee, Shahid Beheshti University of Medical Sciences, Tehran, IR Iran; 2 Department of Epidemiology and Biostatistics, School of Public Health, Tehran University of Medical Sciences, Tehran, IR Iran; 3 Department of Health System Research, Gastroenterology and Liver Disease Research Center, Shahid Beheshti University of Medical Sciences, Tehran, IR Iran

**Keywords:** Hepatitis C, Carcinoma, Hepatocellular, Body Burden

Dear Editor,

We read with interest the paper entitled “Hepatitis C Virus: The Rising Concerns and Growing Hopes, Report From the HCV Symposium, Fourth Tehran Hepatitis Congress, November 2011, Tehran, Iran”, which is written by Alavian et al. (1). This report highlights the rising concerns for future health burden of hepatitis C virus (HCV) in global scale as well as the necessity of allocating HCV awareness issues in order to control HCV in Iranian high risk populations and patients (1). In contrast to Western countries in which alcoholism remains the most common cause of HCC, in Iranian population the Hepatitis B Virus (HBV) and HCV are the main causes of Hepatocellolar Carcinoma (HCC) (2). Fortunately HCV infectionincidence is low in Iran (3) and available data suggest the prevalence of less than 1% in general population (4). Besides, the burden of HCC is low in Iran and it is predicted that the rate of mortality due to this malignancy is leveled off in recent years (5). Although in Iran the mass vaccination program against HBV started in 1993 and reached 94% coverage in 2005 (6), it is supposed that its decreasing impact on the burden of HCC will expose in future decades (5). The epidemiology and prevalence of HCV infection has changed in Iran (7) and studies indicate that prisoners and drug abusers are at high risk of HCV infection (8, 9). Also statistics show that intravenous drug use (IDU) accounts for 50% of HCV transmission routes in our country (1). On the other hand, there is no update information regarding the annually new cases of HCV in general population in order to draw a picture of the trends of HCV infection in Iran and predicting its share in burden of HCC in future. Since we know that the burden of HCC is still too low in our community to predict the role of HBV and HCV in HCC Burden, there is a need to register the annual prevalence of these infections in Iranian population. In the absence of such data, the estimation of burden for HCV, HBV and HCC could be far from the accuracy.

## References

[1] Alavian SM, Jabbari H, Daryani NE, Torabi Nami M (2012). Hepatitis C Virus: The Rising Concerns and Growing Hopes, Report From the HCV Symposium, Fourth Tehran Hepatitis Congress, november 2011, Tehran, Iran.. Hepat Mon..

[2] Fani A, Fani I, Eshrati B, Samadian P, Fani P, Gorishi Y (2007). screening for hepatocellular carcinoma in chronic carriers of hepatitis B and C in markazi province, iran.. Hepat Mon..

[3] Alavian SM, Ahmadzad-Asl M, Lankarani KB, Shahbabaie MA, Bahrami Ahmadi A, Kabir A (2009). Hepatitis C infection in the general population of Iran: a systematic review.. Hepat Mon..

[4] Alavian SM, Adibi P, Zali MR (2005). Hepatitis C virus in Iran: Epidemiology of an emerging infection.. Arch Iranian Med..

[5] Pourhoseingholi MA, Fazeli Z, Zali MR, Alavian SM (2010). Burden of hepatocellular carcinoma in Iran; Bayesian projection and trend analysis.. Asian Pac J Cancer Prev..

[6] Alavian SM, Fallahian F, Lankarani KB (2007). The changing epidemiology of viral hepatitis B in Iran.. J Gastrointestin Liver Dis..

[7] Alavian SM (2009). Hepatitis C infection in Iran; A review article.. Iran J Clin Infect Dis..

[8] Mohtasham Amiri Z, Rezvani M, Jafari Shakib R, Jafari Shakib A (2007). Prevalence of hepatitis C virus infection and risk factors of drug using prisoners in Guilan province.. East Mediterr Health J..

[9] Mohammad Alizadeh AH, Alavian SM, Jafari K, Yazdi N (2005). Prevalence of hepatitis C virus infection and its related risk factors in drug abuser prisoners in Hamedan--Iran.. World J Gastroenterol..

